# MicroRNA in sperm from Duroc, Landrace and Yorkshire boars

**DOI:** 10.1038/srep32954

**Published:** 2016-09-06

**Authors:** Vanmathy Kasimanickam, John Kastelic

**Affiliations:** 1 Veterinary Clinical Sciences Department & Center for Reproductive Biology, College of Veterinary Medicine, Washington State University, Pullman, WA 99164, USA; 2 Department of Production Animal Health, Faculty of Veterinary Medicine, University of Calgary, Calgary, AB T2N 4N2, Canada

## Abstract

Sperm contain microRNAs (miRNAs), which may have roles in epigenetic control. Regarding phylogenetic relationships among various swine breeds, Yorkshire and Landrace, are considered phenotypically and genetically very similar, but distinctly different from Duroc. The objective of the present study was to compare abundance of boar sperm miRNAs in these three breeds. Overall, 252 prioritized miRNAs were investigated using real-time PCR; relative expression of miRNAs in sperm was similar in Yorkshire and Landrace boars, but significantly different compared to Duroc. Seventeen miRNAs (hsa-miR-196a-5p, hsa-miR-514a-3p, hsa-miR-938, hsa-miR-372-3p, hsa-miR-558, hsa-miR-579-3p, hsa-miR-595, hsa-miR-648, hsa-miR-524-3p, hsa-miR-512-3p, hsa-miR-429, hsa-miR-639, hsa-miR-551a, hsa-miR-624-5p, hsa-miR-585-3p, hsa-miR-508-3p and hsa-miR-626) were down-regulated (P < 0.05; fold regulation ≤−2) in Yorkshire and Landrace sperm, compared to Duroc sperm. Furthermore, three miRNAs (hsa-miR-9-5p, hsa-miR-150-5p, and hsa-miR-99a-5p) were significantly up-regulated in Yorkshire and Landrace sperm compared to Duroc sperm, However, 240 miRNAs were not significantly different (within + 2 fold) between Yorkshire and Landrace sperm. We concluded that miRNAs in sperm were not significantly different between Yorkshire and Landrace boars, but there were significant differences between those two breeds and Duroc boars. Furthermore, integrated target genes for selected down-regulated miRNAs (identified via an *in-silico* method) appeared to participate in spermatogenesis and sperm functions.

MicroRNAs are non-coding RNAs that regulate gene expression at a post-transcriptional level and fine-tune expression of ~30% of all mammalian protein-coding genes^1^. Mature miRNAs are single-stranded, with approximately 22 nucleotides^2^. MicroRNA genes are substantially regulated (positively or negatively) by many transcription factors and other proteins, in a tissue- or development-specific manner. Similar to protein-coding genes, microRNA genes are transcribed by RNA polymerase II as large primary transcripts (pri-miRNA) and subsequently processed by RNase III enzyme Drosha to form ~70 nucleotide precursor microRNAs (pre-miRNAs). These pre-miRNAs are subsequently transported to the cytoplasm and processed by RNase III enzyme DICER to form mature miRNAs, which are incorporated into a ribonuclear protein to form a miRNA-induced silencing complex (miRISC) that mediates gene silencing^3^. Post-transcriptional addition of nucleotides to the 3′ ends of pre-miRNAs or mature miRNAs affects miRNA stability or abundance^4^.

Pig domestication has generated several phenotypically distinct breeds, with large differences among breeds for some traits, including reproduction and meat production^5^,^6^,^7^. Although selection for various environments has resulted in a wide variety of domestic pig breeds with apparently divergent phenotypes^8^, genetic variability of miRNA expression, which could be linked to post-transcriptional modifications, has not yet been well characterized. However, a few studies reported differential expression (among various pig breeds) of miRNAs in skeletal muscles^9^, kidneys^10^ and lungs^11^. The present investigation proposed that miRNA expression was not only influenced by stages of development within an individual, types of animal tissues, or age, but also by breed.

Sperm concentration, vitality and motility, as well as semen volume in boars, differed significantly among breeds^12^. For example, Piétrain boars had lower ejaculate volume and total sperm number, but higher sperm concentration than Large White boars^13^,^14^. Furthermore, Piétrain boars produced semen with greater volume and total number of sperm, but lower sperm concentration than Duroc boars^14^,^15^,^16^. Despite these known semen traits differences among breeds, differences in miRNAs have not been well characterized. There are indications that expression of sperm miRNAs were altered by environmental changes^17^ and that sperm traits influenced sperm miRNA expression^18^. A hallmark study^19^ identified robust changes in miRNAs in mouse sperm induced by chronic exposure to stress and suggested that transgenerational epigenetic programming was influenced by sperm miRNAs, thereby providing the impetus to elucidate sperm miRNAs and to distinguish breed specificity with regards to level of expression. The objective of the present study was to determine breed differences in sperm miRNA transcriptome among Landrace, Yorkshire and Duroc boars.

## Materials and Methods

### Ethics statement

This study was performed in strict accordance with standard ethics and use of animal cells for research. The protocol was approved by the institutional animal care and use committee of Washington State University (Protocol Number: 04070-001).

### Boars and semen processing

Fresh boar semen was purchased from a commercial boar semen supplier (Swine Genetics International, Cambridge, IA, USA). All boars were fed similar amounts of a common ration, with nutrition and management in accordance with good industry practices. Semen was collected concurrently from all breeds, with all boars 18 to 24 mo old at the time of semen collection.

Sperm-rich fractions from Yorkshire, Landrace and Duroc boars (n = 6 boars per breed) were used. Initial post-collection motility (subjective microscopic examination) was consistently ≥80%. The sperm-rich fraction was diluted in Beltsville Thawing Solution (1:1 volume) and shipped with gel packs (15 °C) to the laboratory by overnight air freight. Upon arrival, sperm motility was ≥70%, with no evidence of immature sperm or somatic cells, based on a subjective microscopic evaluation. Diluted semen (20 mL) was placed in 50-mL Falcon tubes, centrifuged (1000 × g for 20 min at 4 °C) and seminal plasma separated. Sperm were washed twice using Beltsville Thawing Solution (BTS) at 1000 × g for 20 min at 4 °C. Each sperm pellet was re-suspended in BTS at 4 °C, aliquoted into microcentrifuge tubes (~500 × 10^6^ sperm per tube), and centrifuged at 16,000 × g for 10 min at 4 °C. The supernatant was completely removed and the sperm pellet was flash-frozen in liquid nitrogen and stored at −80 °C until used.

### RNA purification

Total RNA (contains small RNAs, including miRNAs) was isolated from sperm using an RNeasy plus Universal Mini Kit (Qiagen Inc., Valencia, CA, USA), in accordance with manufacturer’s instructions. Briefly, 900 μL QIAzol Lysis Reagent was added to the sperm pellet (~500 × 10^6^ sperm) and thoroughly homogenized using a disposable homogenizer (Thermo Fisher Scientific, San Francisco, CA, USA). The homogenate was placed at room temperature for 5 min to promote dissociation of nucleoprotein complexes. Then, 100 μL of genomic DNA (gDNA) eliminator solution was added and the mixture shaken vigorously to eliminate contamination by gDNA. Chloroform (180 μL) was then added to the homogenate. After vigorous shaking and 2–3 min incubation at room temperature, the mixture was centrifuged at 12,000 × g at 4 °C for 15 min. After centrifugation, the upper aqueous phase (~600 μL) was transferred to a new microcentrifuge tube and 1.5 times volume of 100% ethanol was added. The mixture was mixed thoroughly by repeated pipetting and the sample was centrifuged in an RNeasy mini spin column (8,000 × g for 15 s at room temperature). The RNA was bound to the membrane of the spin column and subsequently removed using buffer RWT and buffer RPE by centrifugation. Thereafter, RNA was eluted in 60 μL RNase-free water. Purity of the RNA was determined using a Thermo Scientific NanoDrop 1000 spectrophotometer; the ratio of absorbance at 260 and 280 nm, respectively, was ~2.0 for all samples. All RNA samples were stored at −80 °C until used.

### Complementary DNA synthesis

Total RNA containing miRNA was used as the starting material. Mature miRNA was reverse-transcribed into cDNA using miScript II RT kit (Qiagen Inc.). Briefly, template RNA was thawed on ice and 10x miScript Nucleics mix, 5x miScript HiSpec buffer and RNase-free water were thawed at room temperature. Reaction components for a 20-μL reaction were 4 μL of HiSpec buffer, 2 μL of Nucleics mix, 2 μL of reverse transcriptase enzyme mix, and 12 μL of RNA template (containing 300 ng RNA plus water). Reverse-transcription reaction components were gently mixed, briefly centrifuged (2000 × g for 10 s) and kept on ice. The mixture was incubated at 37 °C for 60 min and then at 95 °C for 5 min in a Thermocycler (Thermo Fisher Scientific). After incubation, the reaction mix was placed on ice, diluted with 90 μL nuclease-free water, and stored at −20 °C prior to real-time PCR.

### Real-time PCR for sperm mature miRNA profiling

Real-time PCR profiling of sperm mature miRNAs (which eliminated the need for validation as required for microarrays) was performed with miScript miRNA PCR arrays, in combination with the miScript SYBR Green PCR Kit, which contains miScript Universal reverse primer and QuantiTect SYBR Green PCR Master Mix. Human miRNome miScript miRNA PCR array 96-well Plates 1, 2 and 3 (Supplementary Tables 1, 2 and 3) were used, as mature miRNAs are conserved between humans and pigs. A sample of miRNA sequences is shown (Table 1). This array profiled expression of 252 most abundantly expressed and well characterized miRNA sequences in boar sperm (hsa-miR-142-5p to hsa-miR-758-3p; Supplementary Tables 1, 2 and 3). A set of controls in the last row of each plate facilitated data analysis using the ΔΔC_T_ method of relative quantification and for assessing performance of reverse transcription and PCR.

The reaction mix for miScript mature miRNA PCR arrays was prepared with 1375 μL of 2x QuantiTect SYBR Green PCR master mix, 275 μL of 10x miScript universal primer, 1000 μL of RNase free water and 100 μL of template cDNA for each 96-well plate. Furthermore, 25 μL of reaction mix was added to each well and template was amplified in a StepOnePlus Real Time PCR system (Applied Biosystems, Foster City, CA, USA). Cycling conditions were an initial heating step at 95 °C for 15 min. Forty cycles included a 15 s denaturation step at 94 °C, a 30 s annealing step at 55 °C and a 30 s extension step at 70 °C. Dissociation curve analysis was done to verify miRNA specificity and identity.

### Data analysis

Data analysis was performed using a web-based platform (data analysis center web portal; http://www.qiagen.com). The C_T_ values were uploaded and samples were allocated into a control group (Duroc), Group 1 (Yorkshire) and Group 2 (Landrace), using a sample manager for relative comparison of miRNA expression. The gene RNU6-6P was chosen from the house-keeping gene panel to normalize C_T_ values of target miRNAs, since its C_T_ values had <0.5 cycle variation among samples and breeds. The data quality control page was reviewed to ensure that each sample had acceptable PCR array reproducibility, reverse transcription efficiency, and no genomic DNA contamination. After normalization, analyses were performed. The data overview section was examined for each group’s distribution of C_T_ values and the average of the raw data in each group. Average ∆C_T_, 2^∆CT^, Fold Change, and Fold Regulation were calculated and P-values were determined using a Student’s *t*-test (two-tail distribution and equal variances) on the replicates of 2^ΔCT^ values for each miRNA in each breed group, compared to the control breed group. For all analyses, P < 0.05 and fold regulation on relative comparison ≤−2 or ≥ +2 was considered significant differential expression of miRNA. In addition, Yorkshire (control) and Landrace (group 1) were also compared, using the same parameters.

### Integration of target genes

Ten down-regulated miRNAs in comparison groups (Yorkshire/Duroc and Landrace/Duroc) were selected for identification of target genes, which was done with a software algorithm and target mining selection of miRDB (http://mirdb.org/miRDB/mining.html).

## Results and Discussion

**Figure 1:**
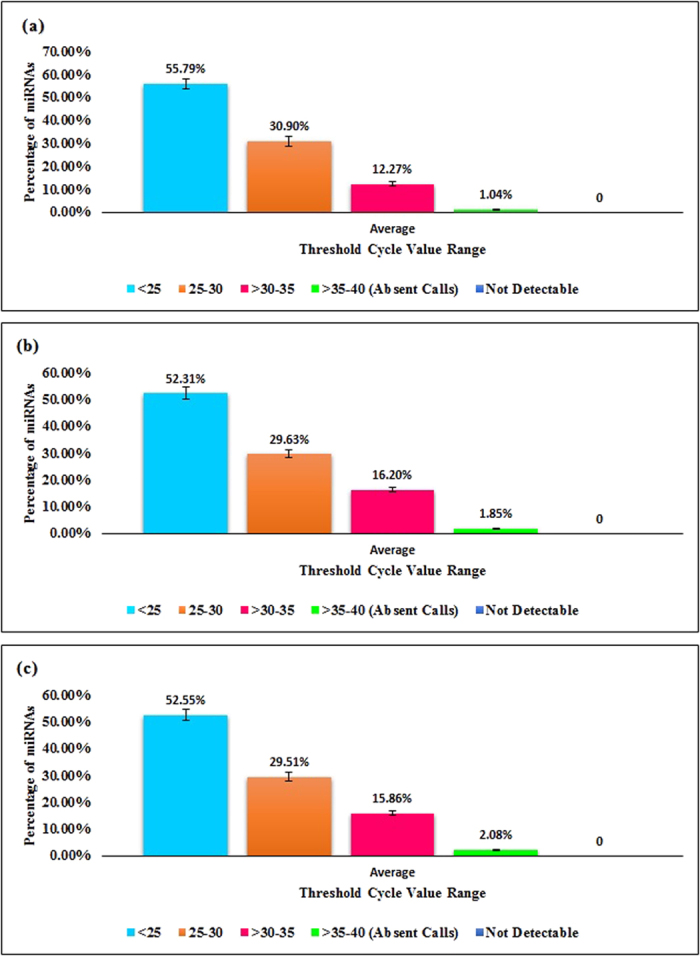
Percent distribution of C_T_ values of boar sperm miRNAs analyzed in three groups [(**a**) control (Duroc), (**b**) Group 1 (Yorkshire) and (**c**) Group 2 (Landrace)]. Note that all miRNAs analyzed were detectable using real-time PCR. The few miRNAs with threshold cycle >35 were omitted from analyses.

The current investigation elucidated sperm miRNAs in Yorkshire, Landrace and Duroc boars. Abundance of MiRNAs from Yorkshire and Landrace sperm were individually compared to Duroc sperm; thereafter, Yorkshire and Landrace were compared. On a genome analysis of cumulative average of nucleotide diversity, Duroc differed significantly from both Yorkshire and Landrace, whereas the latter two breeds had a very close relationship for nucleotide diversity, consistent with being the two closest breeds of pigs based on phylogenetic analyses^20^. In that regard, Yorkshire and Landrace shared components with each other^20^ and based on principal component analyses, could not be clearly distinguished^20^. This was apparently the first study to demonstrate a lack of differential expression of miRNA in sperm between Yorkshire and Landrace boars, as well as expression differences between these two breeds and Durocs. Real-time PCR array reproducibility, reverse transcription control and reverse transcription efficiency were all satisfactory. More than 50% of miRNAs had <25 threshold cycles in the three breed groups analyzed. Approximately 30% miRNAs from the three groups had 25 to 30 cycles and ~16% miRNAs had 31–35 cycles. Furthermore, >35 cycles was considered as absent calls (Fig. 1), with very few miRNAs in this category.

**Figure 2:**
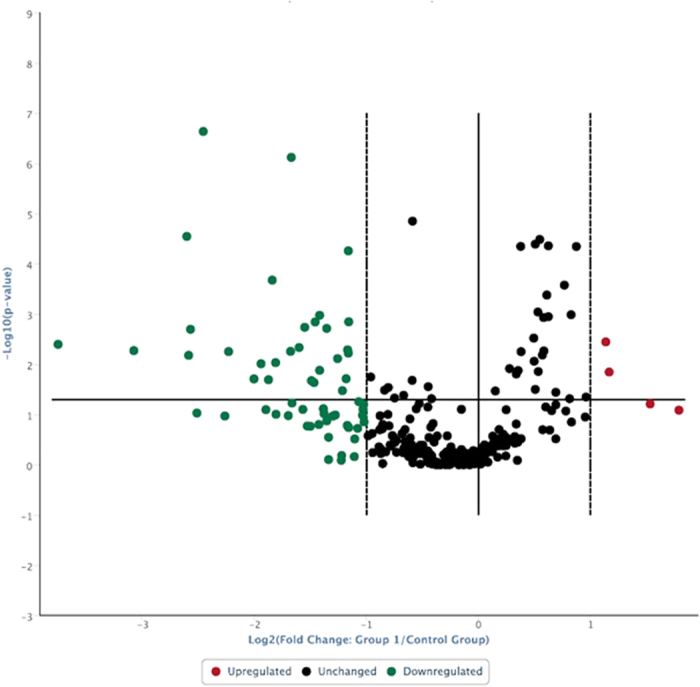
Volcano plot: Log 2 values of relative expression of boar sperm miRNAs (Yorkshire related to Duroc) versus - Log 10 of p-value. Horizontal line is at P = 0.05, whereas vertical lines have been placed at boundary values 2.

**Figure 3:**
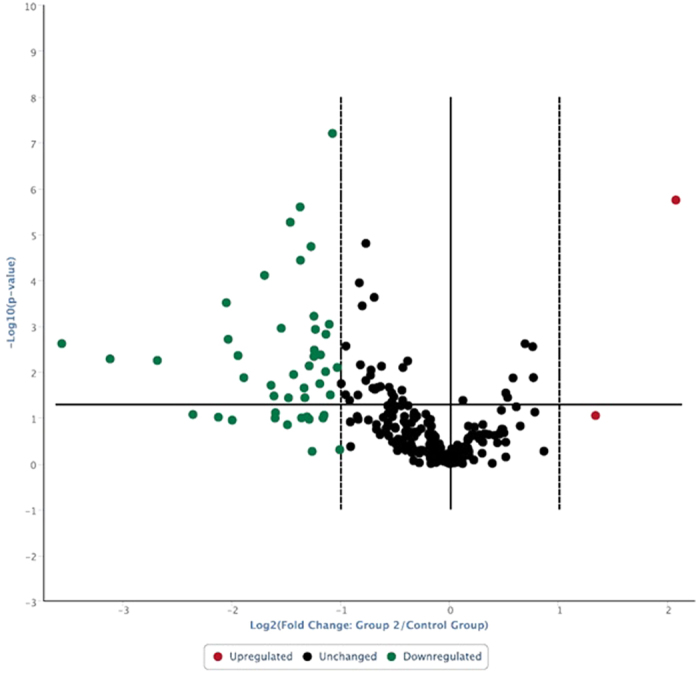
Volcano plot: Log 2 values of relative expression of boar sperm miRNAs (Landrace related to Duroc) versus - Log 10 of p-value. Horizontal line is at P = 0.05, whereas vertical lines have been placed at the boundary value 2.

Out of 252 well-characterized miRNAs investigated in the present study, all were measurably expressed in Duroc, Yorkshire and Landrace sperm. Several miRNA species were down-regulated in Yorkshire and Landrace sperm compared to Duroc sperm, whereas a few miRNAs were up-regulated in Yorkshire and Landrace sperm compared to Duroc sperm (Figs 2 and 3). Furthermore, 27 miRNAs abundances were lower in Yorkshire sperm compared to Duroc sperm (Table 2; <−2 fold regulation and P < 0.05), whereas 30 miRNAs were down-regulated in Landrace boar sperm, compared to Duroc boar sperm (Table 3; <−2 fold regulation and P < 0.05). Seventeen miRNAs (Table 4) were common in significant down-regulation for both comparison groups. Considering fold regulation of +2 as a cut-off, three miRNAs were significantly up-regulated in Yorkshire and Landrace sperm when compared to Duroc sperm (Table 5). It was noteworthy that despite slight variations in miRNA expression between Yorkshire and Landrace, fold regulation of 240 miRNAs did not exceed cut-offs (+ or −2; Table 6). Overall, the 252 miRNAs analyzed in this study were at detectable levels in boar sperm. Although Duroc boars differed from Yorkshire and Landrace boars, the latter two breeds had a close relationship. The down-regulated miRNAs in Yorkshire and Landrace compared to Duroc would have caused up-regulation of genes which may have a role in enhancing desirable semen traits, including greater semen volume, sperm concentration and progressive motility in Yorkshire and Landrace semen compared to Duroc semen. Similar miRNAs expression pattern between Yorkshire and Landrace were consistent with similar quality of semen between these two breeds. Phenotypically similar breeds not only had similar semen traits, but also considerable similarity in sperm miRNA, whereas phenotypically dissimilar breeds differed in semen traits as well as expression of sperm miRNAs. Since all boars used in the study were produced and maintained in the same environment and fed the same ration, differences among breeds in sperm miRNAs were largely attributed to their phenotype and genotype.

Many miRNAs were differentially expressed in mammary gland of lactating Jinhua versus Yorkshire breeds of swine^21^, whereas several miRNAs expressed in the *longissimus dorsi* muscle varied between German Landrace and Pietrain pig breeds^22^. Expression of 125 miRNAs in kidney varied among pig breeds, including the Iberian breed, European Wild Boar ancestor, Landrace, Large White, Piétrain, Meishan and Vietnamese breeds^10^. In skeletal muscle, 54 miRNAs were differentially expressed in Lantang and Landrace pigs and in adipose tissues, the level of 48 miRNAs varied in Lantang and Landrace pigs^9^. Furthermore, expression levels for drug metabolizing genes such as SULT1A1, ABCB1, CYP1A2, CYP2E1, CYP3A22 and CYP3A29 differed among Duroc, Landrace, Yorkshire and Hampshire pigs^23^. In addition, there was higher protein content of Drosha, Dicer and Ago2 (main enzymes required for biogenesis of miRNAs) in the liver of Erhualian versus Large White piglets^24^. Also, semen quality, including sperm concentration, ejaculate volume and sperm number differed among breeds (Large White, Pietrain and Duroc x Pietrain), along with seasonal changes^13^. Estimated overall heritabilities for semen volume and sperm concentration were ~0.20, which is considerable^25^. Specifically, Wolf (2010) calculated the heritability of semen volume as 0.20 +/− 0.019 for Large White and 0.25 +/− 0.018 for Landrace, and the heritability of sperm concentration as 0.18 (SEM = 0.012 and 0.014) in both breeds^26^. On a comparison of boar sperm output among various breeds (Czech Large White, Czech Landrace, Prestice Black-Pied, Czech Meat Pig, Hampshire, Duroc, Pietrain and Large White), Duroc boars had lowest values for sperm quality, whereas Large White had best sperm quality^14^. The present study illustrated differential expression of sperm miRNAs in Yorkshires, Landraces and Durocs. Whereas several other studies have demonstrated that Duroc breed differed genetically from Yorkshire and Landrace breeds for various parameters, the present study identified breed differences and similarities in the context of sperm miRNAs.

Boars have been selected for superior genetics; historically this selection focused on production traits, including age and back fat thickness at 100 kg, feed efficiency, lean yield and litter size^27^. These selection pressures may have negatively affected reproductive traits, including semen quality. However, there is increasing pressure to incorporate male fertility traits, such as sperm number, sperm fertilizing capacity and boar conformation for efficient semen collection. A recent study^28^ recommended that selection indices include four main semen traits, namely volume, concentration, progressive motility, and morphologically abnormal sperm, without compromising genetic benefits from maternal traits and with a minimal loss of genetic gain from paternal traits (other than semen traits) for economic value. Following the current characterization of breed differences in sperm miRNAs, the logical next step would be to determine associations between sperm miRNA abundance and common traits, including progressive motility and fertilizing capacity of sperm. Based on the outcomes, it may be appropriate to use sperm miRNA abundance as a trait in boar selection.

Since the primary function of miRNAs is post-transcriptional regulation of expression of target genes, this study also identified target genes for some down-regulated miRNAs in Yorkshire and Landrace boar sperm, compared to Duroc boar sperm. Target genes with the highest target score (≥90) are shown (Table 7). Several target genes, including SLC9A6, AQP4, EGFR, MAP3K1, NR2C2, PTPRG, RET, ABCB9, PTEN and JAM2^29^,^30^,^31^,^32^,^33^,^34^,^35^,^36^,^37^,^38^ are involved in either spermatogenesis or sperm function. We inferred that these target genes would have been up-regulated in Yorkshire and Landrace sperm, compared to Duroc sperm. Some of these target genes have been associated with spermatogenesis and sperm function. Therefore, down-regulated miRNAs and their target genes presumably contributed to differences in semen quality between phenotypically distinct breeds.

## Conclusions

In summary, all 252 miRNAs analyzed were detected in sperm of Yorkshire, Landrace and Duroc boars, with potential roles in epigenetic regulation of sperm function. Abundance of sperm miRNAs varied among breeds; Duroc boars differed from Yorkshire and Landrace boars, whereas the latter two breeds had a close relationship.

## Additional Information

**How to cite this article**: Kasimanickam, V. and Kastelic, J. MicroRNA in sperm from Duroc, Landrace and Yorkshire boars. *Sci. Rep.* **6**, 32954; doi: 10.1038/srep32954 (2016).

## Supplementary Material

Supplementary Information

## Figures and Tables

**Table 1:** Sample nucleotide sequences of human and porcine mature miRNAs.

miRNA	Nucleotide sequence
hsa-miR-142-5p ssc-miR-142-5p	CAUAAAGUAGAAAGCACUACU CAUAAAGUAGAAAGCACUACU
hsa-miR-9-5p ssc-miR-9-1	UCUUUGGUUAUCUAGCUGUAUGA UCUUUGGUUAUCUAGCUGUAUGA
hsa-miR-150-5p ssc-miR-150	UCUCCCAACCCUUGUACCAGUG UCUCCCAACCCUUGUACCAGUG
hsa-miR-27b-3p ssc-miR-27b-3p	UUCACAGUGGCUAAGUUCUGC UUCACAGUGGCUAAGUUCUGC
hsa-miR-101-3p ssc-miR-101	UACAGUACUGUGAUAACUGAA UACAGUACUGUGAUAACUGAA
hsa-let-7d-5p ssc-let-7d-5p	AGAGGUAGUAGGUUGCAUAGUU AGAGGUAGUAGGUUGCAUAGUU
hsa-miR-103a-3p ssc-miR-103	AGCAGCAUUGUACAGGGCUAUGA AGCAGCAUUGUACAGGGCUAUGA
hsa-miR-16-5p ssc-miR-16	UAGCAGCACGUAAAUAUUGGCG UAGCAGCACGUAAAUAUUGGCG
hsa-miR-26a-5p ssc-miR-26a	UUCAAGUAAUCCAGGAUAGGCU UUCAAGUAAUCCAGGAUAGGCU
hsa-miR-32-5p ssc-miR-32	UAUUGCACAUUACUAAGUUGCA UAUUGCACAUUACUAAGUUGC
hsa-let-7g-5p ssc-let-7g	UGAGGUAGUAGUUUGUACAGUU UGAGGUAGUAGUUUGUACAGUU
hsa-miR-30c-5p ssc-miR-30c-5p	UGUAAACAUCCUACACUCUCAGC UGUAAACAUCCUACACUCUCAGC
hsa-miR-96-5p ssc-miR-96-5p	UUUGGCACUAGCACAUUUUUGCU UUUGGCACUAGCACAUUUUUGCU
hsa-miR-185-5p ssc-miR-185	UGGAGAGAAAGGCAGUUCCUGA UGGAGAGAAAGGCAGUUCCUGA
hsa-miR-142-3p ssc-miR-142-3p	UGUAGUGUUUCCUACUUUAUGGA UGUAGUGUUUCCUACUUUAUGG
hsa-miR-24-3p ssc-miR-24-3p	UGGCUCAGUUCAGCAGGAACAG UGGCUCAGUUCAGCAGGAACAG
hsa-miR-155-5p ssc-miR-155-5p	UUAAUGCUAAUCGUGAUAGGGGU UUAAUGCUAAUUGUGAUAGGGG
hsa-miR-146a-5p ssc-miR-146a-5p	UGAGAACUGAAUUCCAUGGGUU UGAGAACUGAAUUCCAUGGGUU
hsa-miR-425-5p ssc-miR-425-5p	AAUGACACGAUCACUCCCGUUGA AAUGACACGAUCACUCCCGUUGA
hsa-miR-181b-5p ssc-miR-181b	AACAUUCAUUGCUGUCGGUGGGU AACAUUCAUUGCUGUCGGUGGGUU
hsa-miR-30b-5p ssc-miR-30b-5p	UGUAAACAUCCUACACUCAGCU UGUAAACAUCCUACACUCAGCU
hsa-miR-21-5p ssc-miR-21	UAGCUUAUCAGACUGAUGUUGA UAGCUUAUCAGACUGAUGUUGA

Since all mature miRNAs listed are conserved, human miRNome miScript miRNA PCR array 96-well Plates 1, 2 and 3 were used to investigate mature miRNAs in sperm from three breeds of boars.

**Table 2:** Fold regulation of miRNAs in sperm from Yorkshire versus Duroc boars.

Position	miRNA	Fold regulation (Yorkshire/Duroc)	Prob.
2-B04	hsa-miR-196a-5p	−6.01	0.006610
2-B07	hsa-miR-514a-3p	−2.81	0.021147
2-C04	hsa-miR-938	−2.27	0.019341
2-D01	hsa-miR-372-3p	−3.66	0.020250
2-E04	hsa-miR-506-3p	−3.18	0.000001
2-F04	hsa-miR-633	−2.24	0.005143
2-F09	hsa-miR-555	−3.84	0.009713
2-G03	hsa-miR-548b-3p	−2.76	0.023048
2-G10	hsa-miR-184	−2.93	0.001828
3-A02	hsa-miR-558	−3.58	0.000211
3-A03	hsa-miR-579-3p	−4.69	0.005560
3-A04	hsa-miR-595	−3.20	0.005520
3-A08	hsa-miR-648	−6.08	0.000028
3-A09	hsa-miR-206	−2.56	0.001919
3-B05	hsa-miR-524-3p	−3.03	0.004617
3-C05	hsa-miR-512-3p	−2.67	0.013151
3-C10	hsa-miR-422a	−2.67	0.001055
3-D04	hsa-miR-429	−2.23	0.000055
3-D11	hsa-miR-639	−5.94	0.002006
3-D12	hsa-miR-551a	−2.23	0.001423
3-E02	hsa-miR-562	−2.23	0.005978
3-E03	hsa-miR-624-5p	−5.49	0.000000
3-E07	hsa-miR-412-3p	−2.32	0.033384
3-E11	hsa-miR-585-3p	−2.74	0.001431
3-F03	hsa-miR-508-3p	−3.51	0.009239
3-F11	hsa-miR-130b-3p	−2.39	0.007682
3-G11	hsa-miR-626	−4.01	0.019462

MiRNAs that had <−2 fold regulation in related groups (Yorkshire/Duroc) and were P < 0.05 (Student’s *t*-test of replicates of 2^ΔCt^ values).

**Table 3:** Fold regulation of miRNAs in sperm from Landrace versus Duroc boars.

Position	miRNA	Fold regulation (Landrace/Duroc)	Prob
1-F03	hsa-miR-302a-3p	−2.38	0.000606
2-A05	hsa-miR-376b-3p	−2.45	0.007334
2-B04	hsa-miR-196a-5p	−3.71	0.013271
2-B05	hsa-miR-658	−3.26	0.000078
2-B07	hsa-miR-514a-3p	−2.60	0.000003
2-C04	hsa-miR-938	−2.20	0.001496
2-C09	hsa-miR-370-3p	−2.42	0.000018
2-D01	hsa-miR-372-3p	−2.71	0.011340
2-D11	hsa-miR-371a-3p	−2.36	0.001171
2-E11	hsa-miR-563	−2.80	0.036534
2-F02	hsa-miR-621	−2.37	0.003330
3-A02	hsa-miR-558	−3.12	0.019469
3-A03	hsa-miR-579-3p	−11.82	0.002401
3-A04	hsa-miR-595	−2.28	0.004212
3-A06	hsa-miR-542-5p	−2.29	0.018017
3-A08	hsa-miR-648	−4.15	0.000308
3-B02	hsa-miR-559	−2.16	0.000910
3-B03	hsa-miR-369-5p	−2.11	0.000000
3-B04	hsa-miR-484	−2.38	0.004573
3-B05	hsa-miR-524-3p	−2.53	0.022224
3-C05	hsa-miR-512-3p	−2.14	0.031518
3-C11	hsa-miR-635	−2.52	0.036319
3-D04	hsa-miR-429	−2.76	0.000005
3-D11	hsa-miR-639	−4.10	0.001937
3-D12	hsa-miR-551a	−2.59	0.000036
3-E03	hsa-miR-624-5p	−3.07	0.033279
3-E08	hsa-miR-566	−2.05	0.007957
3-E11	hsa-miR-585-3p	−2.93	0.001110
3-F03	hsa-miR-508-3p	−2.21	0.009850
3-G11	hsa-miR-626	−3.85	0.004368

MiRNAs that had <−2 fold regulation in related groups (Landrace/Duroc) and were P < 0.05 (Student’s *t*-test of replicates of 2^ΔCt^ values).

**Table 4:** Fold regulation of miRNAs in sperm from Yorkshire and Landrace versus Duroc boars.

Position	miRNA	Fold regulation (Yorkshire/Duroc)	Prob.	Fold regulation (Landrace/Duroc)	Prob.
2-B04	hsa-miR-196a-5p	−6.01	0.006610	−3.71	0.013271
2-B07	hsa-miR-514a-3p	−2.81	0.021147	−2.60	0.000003
2-C04	hsa-miR-938	−2.27	0.019341	−2.20	0.001496
2-D01	hsa-miR-372-3p	−3.66	0.020250	−2.71	0.011340
3-A02	hsa-miR-558	−3.58	0.000211	−3.12	0.019469
3-A03	hsa-miR-579-3p	−4.69	0.005560	−11.82	0.002401
3-A04	hsa-miR-595	−3.20	0.005520	−2.28	0.004212
3-A08	hsa-miR-648	−6.08	0.000028	−4.15	0.000308
3-B05	hsa-miR-524-3p	−3.03	0.004617	−2.53	0.022224
3-C05	hsa-miR-512-3p	−2.14	0.031518	−2.14	0.031518
3-D04	hsa-miR-429	−2.23	0.000055	−2.76	0.000005
3-D11	hsa-miR-639	−5.94	0.002006	−4.10	0.001937
3-D12	hsa-miR-551a	−2.23	0.001423	−2.59	0.000036
3-E03	hsa-miR-624-5p	−5.49	0.000000	−3.07	0.033279
3-E11	hsa-miR-585-3p	−2.74	0.001431	−2.93	0.001110
3-F03	hsa-miR-508-3p	−3.51	0.009239	−2.21	0.009850
3-G11	hsa-miR-626	−4.01	0.019462	−3.85	0.004368

MiRNAs that had <−2 fold regulation in both related groups (Yorkshire/Duroc and Landrace/Duroc) and were P < 0.05 (Student’s *t*-test of replicates of 2^ΔCt^ values).

**Table 5:** Fold regulation of miRNAs in sperm from Yorkshire and Landrace versus Duroc boars.

Position	miRNA	Fold regulation (Yorkshire/Duroc)	Prob.
1-A02	hsa-miR-9-5p	2.20	0.003577
1-A03	hsa-miR-150-5p	2.25	0.014231
**Position**	**miRNA**	**Fold regulation (Landrace/Duroc)**	**p value**
1-C10	hsa-miR-99a-5p	4.20	0.000002

MiRNAs that had >2 fold regulation in either of related groups (Yorkshire/Duroc and Landrace/Duroc) and were P < 0.05 (Student’s *t*-test of the replica*t*es of 2^ΔCt^ values).

**Table 6:** Fold Regulation of miRNAs in sperm from Landrace versus Yorkshire boars.

Position	miRNA	Fold regulation (Landrace/Yorkshire)	Prob.
1-A02	hsa-miR-9-5p	−2.1435	0.005045
1-A03	hsa-miR-150-5p	−1.4777	0.185451
1-C10	hsa-miR-99a-5p	1.6634	0.200543
1-F03	hsa-miR-302a-3p	−1.234	0.369133
2-A05	hsa-miR-376b-3p	1.0644	0.901582
2-B04	hsa-miR-196a-5p	1.617	0.054369
2-B05	hsa-miR-658	−1.2142	0.321848
2-B06	hsa-miR-511-5p	1.1303	0.929117
2-B07	hsa-miR-514a-3p	1.0817	0.401646
2-C04	hsa-miR-938	1.0281	0.880384
2-C09	hsa-miR-370-3p	−1.4241	0.229018
2-D01	hsa-miR-372-3p	1.3535	0.957798
2-D11	hsa-miR-371a-3p	−1.544	0.096848
2-E04	hsa-miR-506-3p	2.0373	0.048769
2-E11	hsa-miR-563	1.0116	0.600244
2-F02	hsa-miR-621	−1.7492	0.139796
2-F04	hsa-miR-633	1.9816	0.117439
2-F09	hsa-miR-555	1.9725	0.48651
2-G03	hsa-miR-548b-3p	1.8618	0.303309
2-G10	hsa-miR-184	3.088	0.001853
3-A02	hsa-miR-558	1.146	0.407224
3-A03	hsa-miR-579-3p	−2.5198	0.00401
3-A04	hsa-miR-595	1.4012	0.687305
3-A06	hsa-miR-542-5p	−1.4306	0.112653
3-A08	hsa-miR-648	1.4641	0.494697
3-A09	hsa-miR-206	3.5738	0.000013
3-B02	hsa-miR-559	−1.0305	0.551599
3-B03	hsa-miR-369-5p	−1.1701	0.34168
3-B04	hsa-miR-484	−1.3134	0.337444
3-B05	hsa-miR-524-3p	1.1975	0.698579
3-C05	hsa-miR-512-3p	1.2454	0.679649
3-C10	hsa-miR-422a	1.6096	0.199596
3-C11	hsa-miR-635	−1.4208	0.16004
3-D04	hsa-miR-429	−1.2368	0.203141
3-D11	hsa-miR-639	1.4473	0.641879
3-D12	hsa-miR-551a	−1.162	0.526134
3-E02	hsa-miR-562	1.2426	0.639007
3-E03	hsa-miR-624-5p	1.7901	0.177284
3-E07	hsa-miR-412-3p	1.4506	0.463352
3-E08	hsa-miR-566	−1.1147	0.609148
3-E11	hsa-miR-585-3p	−1.0668	0.840865
3-F03	hsa-miR-508-3p	1.5874	0.489521
3-F11	hsa-miR-130b-3p	1.6187	0.073362
3-G11	hsa-miR-626	1.0401	0.861144

Differential expression of MiRNAs were detected, but were not significant (except four miRNAs) based on fold regulation and P-value (the latter was calculated based on Student’s t-test of the replicates of 2^ΔCt^ values).

**Table 7:** Target genes for the first 10 down-regulated boar sperm miRNAs in both comparison groups are shown, using target mining selection of miRDB (http://mirdb.org/miRDB/mining.html).

miRNA	Gene symbol	Gene description	Target score
hsa-miR-196a-5p	ZMYND11	zinc finger, MYND-type containing 11	100
hsa-miR-196a-5p	SLC9A6	solute carrier family 9, subfamily A (NHE6, cation proton antiporter 6), member 6	100
hsa-miR-196a-5p	AQP4	aquaporin 4	98
hsa-miR-514a-3p	EGFR	epidermal growth factor receptor	98
hsa-miR-514a-3p	CABLES1	Cdk5 and Abl enzyme substrate 1	98
hsa-miR-196a-5p	HOXB7	homeobox B7	97
hsa-miR-514a-3p	AGO4	argonaute RISC catalytic component 4	97
hsa-miR-196a-5p	NR2C2	nuclear receptor subfamily 2, group C, member 2	97
hsa-miR-514a-3p	ECE1	endothelin converting enzyme 1	97
hsa-miR-196a-5p	GATA6	GATA binding protein 6	96
hsa-miR-514a-3p	NCOA7	nuclear receptor coactivator 7	96
hsa-miR-514a-3p	PTEN	phosphatase and tensin homolog	96
hsa-miR-196a-5p	PBX1	pre-B-cell leukemia homeobox 1	96
hsa-miR-514a-3p	QSER1	glutamine and serine rich 1	95
hsa-miR-196a-5p	ERI2	ERI1 exoribonuclease family member 2	95
hsa-miR-514a-3p	JAM2	junctional adhesion molecule 2	95
hsa-miR-514a-3p	C7	complement component 7	95
hsa-miR-196a-5p	DENND6A	DENN/MADD domain containing 6A	94
hsa-miR-196a-5p	CCDC47	coiled-coil domain containing 47	94
hsa-miR-514a-3p	BAALC	brain and acute leukemia, cytoplasmic	94
hsa-miR-196a-5p	RDX	Radixin	94
hsa-miR-196a-5p	MAP3K1	mitogen-activated protein kinase kinase kinase 1, E3 ubiquitin protein ligase	94
hsa-miR-514a-3p	TMEM68	transmembrane protein 68	94
hsa-miR-514a-3p	SLC39A9	solute carrier family 39, member 9	94
hsa-miR-196a-5p	HOXC8	homeobox C8	94
hsa-miR-196a-5p	CEP350	centrosomal protein 350kDa	93
hsa-miR-514a-3p	SPTLC3	serine palmitoyltransferase, long chain base subunit 3	93
hsa-miR-514a-3p	AFF4	AF4/FMR2 family, member 4	93
hsa-miR-514a-3p	PTPRG	protein tyrosine phosphatase, receptor type, G	93
hsa-miR-196a-5p	ELAVL4	ELAV like neuron-specific RNA binding protein 4	93
hsa-miR-514a-3p	COL2A1	collagen, type II, alpha 1	93
hsa-miR-196a-5p	ABCB9	ATP-binding cassette, sub-family B (MDR/TAP), member 9	93
hsa-miR-514a-3p	TCF12	transcription factor 12	92
hsa-miR-196a-5p	RCC2	regulator of chromosome condensation 2	92
hsa-miR-196a-5p	HOXA7	homeobox A7	92
hsa-miR-514a-3p	SYT11	synaptotagmin XI	92
hsa-miR-196a-5p	NTN4	netrin 4	92
hsa-miR-196a-5p	HOXA5	homeobox A5	92
hsa-miR-514a-3p	ZNF282	zinc finger protein 282	91
hsa-miR-514a-3p	USP27X	ubiquitin specific peptidase 27, X-linked	91
hsa-miR-514a-3p	FAM117A	family with sequence similarity 117, member A	91
hsa-miR-196a-5p	CCNJ	cyclin J	91
hsa-miR-514a-3p	ZNF800	zinc finger protein 800	91
hsa-miR-196a-5p	NR6A1	nuclear receptor subfamily 6, group A, member 1	91
hsa-miR-514a-3p	PLCL1	phospholipase C-like 1	91
hsa-miR-196a-5p	SMC3	structural maintenance of chromosomes 3	90
hsa-miR-196a-5p	LARP4	La ribonucleoprotein domain family, member 4	90
hsa-miR-196a-5p	TSTD3	thiosulfate sulfurtransferase (rhodanese)-like domain containing 3	90
hsa-miR-196a-5p	LRIG3	leucine-rich repeats and immunoglobulin-like domains 3	90
hsa-miR-514a-3p	PPP2R1A	protein phosphatase 2, regulatory subunit A, alpha	90
hsa-miR-196a-5p	PACRGL	PARK2 co-regulated-like	90
hsa-miR-196a-5p	ZNF850	zinc finger protein 850	90
hsa-miR-196a-5p	TMX1	thioredoxin-related transmembrane protein 1	90
hsa-miR-196a-5p	LIX1L	Lix1 homolog (mouse)-like	90
hsa-miR-514a-3p	PCCA	propionyl CoA carboxylase, alpha polypeptide	90
hsa-miR-514a-3p	AKAP10	A kinase (PRKA) anchor protein 10	90
hsa-miR-514a-3p	NCOR1	nuclear receptor corepressor 1	90
hsa-miR-196a-5p	LRIG2	leucine-rich repeats and immunoglobulin-like domains 2	90
hsa-miR-196a-5p	RET	ret proto-oncogene	90

Only functional miRNAs were included. MiRNAs with >300 predicted targets in genome were excluded, whereas target genes with ≥ 90 target score were shown.
