# Supplementary material for: MicroRNA in sperm from Duroc, Landrace and Yorkshire boars
Source: Sci Rep. 2016 Sep 6;6:32954. doi: 10.1038/srep32954 (PMC5011730; doi:10.1038/srep32954)
Supplement: Supplementary Information [file srep32954-s1.pdf]

Title: MicroRNA in sperm from Duroc, Landrace and Yorkshire boars

Authors: Vanmathy Kasimanickam & John Kastelic

96	1	2	3	4	5	6	7	8	9	10	11	12
A	hsa-miR-142-5p	hsa-miR-9-5p	hsa-miR-150-5p	hsa-miR-27b-3p	hsa-miR-101-3p	hsa-let-7d-5p	hsa-miR-103a-3p	hsa-miR-16-5p	hsa-miR-26a-5p	hsa-miR-32-5p	hsa-miR-26b-5p	hsa-let-7g-5p
B	hsa-miR-30c-5p	hsa-miR-96-5p	hsa-miR-185-5p	hsa-miR-142-3p	hsa-miR-24-3p	hsa-miR-155-5p	hsa-miR-146a-5p	hsa-miR-425-5p	hsa-miR-181b-5p	hsa-miR-302b-3p	hsa-miR-30b-5p	hsa-miR-21-5p
C	hsa-miR-30e-5p	hsa-miR-200c-3p	hsa-miR-15b-5p	hsa-miR-223-3p	hsa-miR-194-5p	hsa-miR-210-3p	hsa-miR-15a-5p	hsa-miR-181a-5p	hsa-miR-125b-5p	hsa-miR-99a-5p	hsa-miR-28-5p	hsa-miR-320a
D	hsa-miR-125a-5p	hsa-miR-29b-3p	hsa-miR-29a-3p	hsa-miR-141-3p	hsa-miR-19a-3p	hsa-miR-18a-5p	hsa-miR-374a-5p	hsa-miR-423-5p	hsa-let-7a-5p	hsa-miR-124-3p	hsa-miR-92a-3p	hsa-miR-23a-3p
E	hsa-miR-25-3p	hsa-let-7e-5p	hsa-miR-376c-3p	hsa-miR-126-3p	hsa-miR-144-3p	hsa-miR-424-5p	hsa-miR-30a-5p	hsa-miR-23b-3p	hsa-miR-151a-5p	hsa-miR-195-5p	hsa-miR-143-3p	hsa-miR-30d-5p
F	hsa-miR-191-5p	hsa-let-7i-5p	hsa-miR-302a-3p	hsa-miR-222-3p	hsa-let-7b-5p	hsa-miR-19b-3p	hsa-miR-17-5p	hsa-miR-93-5p	hsa-miR-186-5p	hsa-miR-196b-5p	hsa-miR-27a-3p	hsa-miR-22-3p
G	hsa-miR-130a-3p	hsa-let-7c-5p	hsa-miR-29c-3p	hsa-miR-140-3p	hsa-miR-128-3p	hsa-let-7f-5p	hsa-miR-122-5p	hsa-miR-20a-5p	hsa-miR-106b-5p	hsa-miR-7-5p	hsa-miR-100-5p	hsa-miR-302c-3p
H	cel-miR-39-3p	cel-miR-39-3p	SNORD61	SNORD68	SNORD72	SNORD95	SNORD96A	RNU6-6P	miRTC	miRTC	PPC	PPC

Supplementary Table 1: MicroRNAs in Plate 1; Rows A to G contained specific primers for target microRNAs; and Row H consisted of control genes (including reference microRNAs).

96	1	2	3	4	5	6	7	8	9	10	11	12
A	hsa-miR-346	hsa-miR-214-3p	hsa-miR-643	hsa-miR-608	hsa-miR-376b-3p	hsa-miR-944	hsa-miR-211-5p	hsa-miR-612	hsa-miR-411-5p	hsa-miR-548c-3p	hsa-miR-299-3p	hsa-miR-523-3p
B	hsa-miR-382-5p	hsa-miR-515-3p	hsa-miR-375	hsa-miR-196a-5p	hsa-miR-658	hsa-miR-511-5p	hsa-miR-514a-3p	hsa-miR-659-3p	hsa-miR-597-5p	hsa-miR-652-3p	hsa-miR-183-5p	hsa-miR-192-5p
C	hsa-miR-421	hsa-miR-512-5p	hsa-miR-410-3p	hsa-miR-938	hsa-miR-641	hsa-miR-628-3p	hsa-miR-188-5p	hsa-miR-181c-5p	hsa-miR-370-3p	hsa-miR-301b-3p	hsa-miR-340-5p	hsa-miR-330-3p
D	hsa-miR-372-3p	hsa-miR-379-5p	hsa-miR-10a-5p	hsa-miR-193b-3p	hsa-miR-651-5p	hsa-miR-618	hsa-miR-518a-3p	hsa-miR-647	hsa-miR-564	hsa-miR-548d-3p	hsa-miR-371a-3p	hsa-miR-190a-5p
E	hsa-miR-33a-5p	hsa-miR-504-5p	hsa-miR-18b-5p	hsa-miR-506-3p	hsa-miR-324-5p	hsa-miR-215-5p	hsa-miR-95-3p	hsa-miR-376a-3p	hsa-miR-770-5p	hsa-miR-532-5p	hsa-miR-563	hsa-miR-433-3p
F	hsa-miR-501-5p	hsa-miR-621	hsa-miR-505-3p	hsa-miR-633	hsa-miR-744-5p	hsa-miR-581	hsa-miR-10b-5p	hsa-miR-576-5p	hsa-miR-555	hsa-miR-605-5p	hsa-miR-520d-3p	hsa-miR-455-5p
G	hsa-miR-497-5p	hsa-miR-646	hsa-miR-548b-3p	hsa-miR-522-3p	hsa-miR-575	hsa-miR-187-3p	hsa-miR-615-3p	hsa-miR-365a-3p	hsa-miR-325	hsa-miR-184	hsa-miR-660-5p	hsa-miR-148b-3p
H	cel-miR-39-3p	cel-miR-39-3p	SNORD61	SNORD68	SNORD72	SNORD95	SNORD96A	RNU6-6P	miRTC	miRTC	PPC	PPC

Supplementary Table 2: MicroRNAs in Plate 2; Rows A to G contained specific primers for target microRNAs; and Row H consisted of control genes (including reference microRNAs).

96	1	2	3	4	5	6	7	8	9	10	11	12
A	hsa-miR-542-3p	hsa-miR-558	hsa-miR-579-3p	hsa-miR-595	hsa-miR-362-5p	hsa-miR-542-5p	hsa-miR-200a-5p	hsa-miR-648	hsa-miR-206	hsa-miR-363-3p	hsa-miR-148a-3p	hsa-miR-650
B	hsa-miR-132-3p	hsa-miR-559	hsa-miR-369-5p	hsa-miR-484	hsa-miR-524-3p	hsa-miR-616-5p	hsa-miR-423-3p	hsa-miR-556-5p	hsa-miR-619-3p	hsa-miR-622	hsa-miR-582-5p	hsa-miR-549a
C	hsa-miR-486-5p	hsa-miR-20b-5p	hsa-miR-630	hsa-miR-525-3p	hsa-miR-512-3p	hsa-miR-384	hsa-miR-149-5p	hsa-miR-146b-5p	hsa-miR-487b-3p	hsa-miR-422a	hsa-miR-635	hsa-miR-152-3p
D	hsa-miR-450a-5p	hsa-miR-212-3p	hsa-miR-361-5p	hsa-miR-429	hsa-miR-208a-3p	hsa-miR-487a-3p	hsa-miR-488-3p	hsa-miR-449a	hsa-miR-649	hsa-miR-203a-3p	hsa-miR-639	hsa-miR-551a
E	hsa-miR-769-5p	hsa-miR-562	hsa-miR-624-5p	hsa-miR-610	hsa-miR-127-3p	hsa-miR-134-5p	hsa-miR-412-3p	hsa-miR-566	hsa-miR-492	hsa-miR-518b	hsa-miR-585-3p	hsa-miR-34a-5p
F	hsa-miR-502-5p	hsa-miR-452-5p	hsa-miR-508-3p	hsa-miR-607	hsa-miR-133b	hsa-miR-766-3p	hsa-miR-600	hsa-miR-202-3p	hsa-miR-583	hsa-miR-448	hsa-miR-130b-3p	hsa-miR-454-3p
G	hsa-miR-524-5p	hsa-miR-182-5p	hsa-miR-495-3p	hsa-miR-335-5p	hsa-miR-135b-5p	hsa-miR-34c-5p	hsa-miR-1-3p	hsa-miR-662	hsa-miR-942-5p	hsa-miR-591	hsa-miR-626	hsa-miR-758-3p
H	cel-miR-39-3p	cel-miR-39-3p	SNORD61	SNORD68	SNORD72	SNORD95	SNORD96A	RNU6-6P	miRTC	miRTC	PPC	PPC

Supplementary Table 3: MicroRNAs in Plate 3; Rows A to G contained specific primers for target microRNAs; and Row H consisted of control genes (including reference microRNAs).
